# How many years does it take to establish a specialty in emergency medicine?

**DOI:** 10.1186/1757-7241-23-S1-A50

**Published:** 2015-07-16

**Authors:** Peter Hallas, Lars Folkestad, Dan B Pedersen, Mikkel Brabrand

**Affiliations:** 1 Department of pediatric anesthesia 4013, Rigshospitalet, Copenhagen, Denmark; 2 Endokrinologisk afdeling M, OUH Odense Universitetshospital, Odense, Denmark; 3 Dansk Selskab For Akut Medicin (DASEM), Aarhus, Denmark; 4 Fælles Akutmodtagelse FAM, OUH Odense Universitetshospital, Odense, Denmark; 5 Fælles Akutmodtagelse FAM, Sydvestjysk Sygehus, Esbjerg, Denmark

## Background

Establishing a specialty in Emergency Medicine (EM) is an important step in improving emergency care. The aim of the present study is to identify how long it takes to establish an EM specialty in a European country.

## Methods

An online questionnaire was sent to the national EM societies listed as members of the European Society for Emergency Medicine (EuSEM) and Iceland. The EM societies were contacted via public contact email-addresses and via the EuSEM office. Data from 31 countries were sought. The questionnaire housed three questions: 1) *"What year was your national society in Emergency Medicine founded?" *2) *"What year was EM recognized as a supra- or subspecialty?" *3) *"What year was EM recognized as a specialty (in its own right)?" *Fisher's exact test and t-test was used for analyses.

## Results

We received 17 answers (response rate of 61%). The majority (65%, n = 11) had an established specialty in EM. The overall time from the Foundation of a national society until a specialty was recognized was six years. In 52% (n = 9) of the countries, EM was or had been a supraspecialty or equivalent. Having a supraspecialty (or equivalent) in EM did not affect the likelihood of having a specialty later on (p = 1.00). The age of the national EM society did not differ between countries with and those without a specialty (13 versus 9 years; p > 0.05). For countries with a *supraspecialty or equivalent*, it took 7 years to have an EM specialty in its own right, from the foundation of an EM society. For countries *without a supraspecialty or equivalent*, it took 4.4 years, from the foundation of an EM society to have a specialty in EM recognized. Establishing *a supraspecialty or equivalent *increased the time to an EM specialty in its own right significantly (p > 0.05).

## Conclusions

In the European countries, it takes about 6 years on average from forming of a national society for emergency medicine to the formal recognition of a specialty albeit with large variations. Starting out with a supraspecialty in EM (or equivalent) may not increase the likelihood of an EM specialty.

